# Evaluation of the Antidiabetic and Insulin Releasing Effects of *A. squamosa*, Including Isolation and Characterization of Active Phytochemicals

**DOI:** 10.3390/plants9101348

**Published:** 2020-10-12

**Authors:** Prawej Ansari, Peter R. Flatt, Patrick Harriott, Yasser H.A. Abdel-Wahab

**Affiliations:** School of Biomedical Sciences, Ulster University, Co. Londonderry, Coleraine BT52 1SA, NI, UK; pr.flatt@ulster.ac.uk (P.R.F.); p.harriott@ulster.ac.uk (P.H.); y.abdel-wahab@ulster.ac.uk (Y.H.A.A.-W.)

**Keywords:** diabetes, phytochemicals, glucose, insulin, DPP-IV

## Abstract

*Annona squamosa* is generally referred to as a ‘custard apple’. Antidiabetic actions of hot water extract of *Annona squamosa* (HWAS) leaves together with isolation of active insulinotropic compounds were studied. Insulin release, membrane potential and intracellular Ca^2+^ were determined using BRIN-BD11 cells and isolated mouse islets. 3T3L1 adipocytes and in vitro models were used to determine cellular glucose uptake, insulin action, starch digestion, glucose diffusion, DPP-IV activity and glycation. Glucose intolerant high-fat fed rats were used for in vivo studies. Active compounds were isolated and characterized by HPLC, LCMS and NMR. HWAS stimulated insulin release from clonal β-cells and mouse islets. Using fluorescent indicator dyes and modulators of insulin secretion, effects could be attributed to depolarization of β-cells and influx of Ca^2+^. Secretion was stimulated by isobutylmethylxanthine (IBMX), tolbutamide or 30 mM KCl, indicating additional non-K_ATP_ dependent pathways. Extract stimulated cellular glucose uptake and insulin action and inhibited starch digestion, protein glycation, DPP-IV enzyme activity and glucose diffusion. Oral HWAS improved glucose tolerance and plasma insulin in high-fat fed obese rats. Treatment for 9 days with HWAS (250 mg/5 mL/kg), partially normalised energy intake, body weight, pancreatic insulin content, and both islet size and beta cell mass. This was associated with improved oral glucose tolerance, increased plasma insulin and inhibition of plasma DPP-IV activity. Isolated insulinotropic compounds, including rutin (C_27_H_30_O_16_), recapitulated the positive actions of HWAS on beta cells and in vivo glucose tolerance and plasma insulin responses. *Annona squamosa* is attractive as a dietary adjunct in treatment of T2DM and as a source of potential antidiabetic agents including rutin.

## 1. Introduction

Around 2.8% of the world’s population has diabetes mellitus and it is anticipated to increase to 5.4% of the disease representing 90–95% of the total number of cases [1]. In type 2 diabetes mellitus (T2DM), pancreatic β-cell function is impaired and insulin resistance occurs in hepatic and peripheral tissues, resulting in unrestrained hepatic glucose output and impaired peripheral glucose uptake [2]. Oral hypoglyceamic agents (OHAs) can reduce insulin resistance and facilitate insulin secretion during the early stage of the disease [3,4]. However, more intensive therapy increases the risk of developing gastrointestinal disturbances, weight gain, diarrhea, renal failure and hypoglycaemia [5]. In many countries, such drugs are not freely available, so one avenue to combat T2DM is to use natural sources such as medicinal plants and animal products [6,7].

Herbal medicines have been valuable source of pharmacological therapeutics for diabetes that predates the discovery of insulin and other pharmacological treatments. In an attempt to develop more effective and adjunctive therapy, they have become a growing part of modern medicine [8]. Different plants have been studied in recent times to evaluate their antihyperglycaemic properties [6]. The active phytoconstituents with insulinotropic, insulin mimetic or glucose lowering action of these plants have been also investigated [9]. The anti-hyperglyceamic activity is mainly due to their ability to restore endocrine pancreatic function by increasing the insulin output, inhibiting the intestinal glucose absorption and/or facilitating insulin action [10]. To date, more than 400 plant species have been identified having hypoglyceamic activity, and yet new compounds are being searched for greater efficacy [11].

*Annona squamosa*, locally known as ‘Ata’ belongs to the Annonaceae family and is a small tree native to Bangladesh. The plant is well known locally for different therapeutic attributes such as antioxidant, antidiabetic and hepatoprotective activity [12]. Several phytochemicals have been isolated from different fractions of *A. squamosa* including alkaloids, terpenoids, phenolics, wax and fats, kaempferol, farmarixetin, tannins, flavonoids and steroids [13,14]. Several animal models studies using *A. squamosa* have shown reduction of total cholesterol, LDL and triglyceride, but increased HDL [15,16]. *A. squamosa* also appeared to have the ability to reduce blood glucose and increase plasma insulin levels [17] and to protect from pancreatic β-cells from damage due to oxidative stress [18,19,20,21]. In addition, the plant has been reported to have hepatoprotective properties. Similarly, another study reported *A. squamosa* extract reduced liver toxicity induced by isoniazid plus rifampicin [22]. Other notable pharmacological properties reported include anti-depressant, analgesic, anti-inflammatory [23] and anti-bacterial effects [23].

Despite these studies, the full mechanism of action of *A. squamosa* is unknown and, the present study investigated a broad spectrum of actions of *A. squamosa* as a possible anti-diabetic agent. This included evaluation of effects of hot water extract of *A. squamosa* leaves on insulin secretion, cellular glucose uptake, starch digestion, glucose diffusion, DPP-IV activity and protein glycation. Furthermore, the acute and long-term effects of *A. squamosa* in vivo were evaluated in normal and high-fat-fed diabetic rats. Rats were treated with *A. squamosa* orally (250 mg/5 mL/kg body weight) for 9 days. Oral glucose tolerance, plasma DPP-IV activity, islet morphology and body weight were investigated. Finally, the active compounds from *A. squamosa* extract responsible for increased insulin secretion were isolated using RP-HPLC and their mass to charge ratio and identity were defined by NMR.

## 2. Materials and Methods

### 2.1. Collection and Preparation of Plant Extracts

*Annona squamosa* leaves were obtained from the University Ayurvedic Research Centre (UARC), Jahangirnagar University, Dhaka, Bangladesh. The plant material was identified and documented by the botanical taxonomist Bangladesh National Herbarium, Mirpur, Dhaka, and given accession number 43754. Plant leaves were washed and air-dried, before proceeding with hot water extraction. Twenty-five grams of the dried powder of leaves were added to 1 L of water and heated. When it reached the boiling point, the blend was allowed to stand for 15 min prior to separation using filter paper (Whatman no. 1 filter paper). The oily-separated solution was then dried under a vacuum (Savant Speed vac; New York, NY, USA) to leave a final sticky residue of the plant extract. This was collected and stored at 4 °C until the bioassay was performed [24].

### 2.2. In Vitro Insulin-Releasing Studies

The insulin-releasing effects of plant extract were tested using BRIN-BD11 cells and isolated mouse islets as described previously [11]. A range of concentrations of plant extract or known modulators of insulin secretion were incubated with BRIN-BD11 cells in the presence or absence of glucose (1.1, 5.6 or 16.7 mM) during 20 min incubation at 37 °C. Islets were isolated from the pancreas of albino Swiss mice (40–50 gm) by digesting with collagenase P from *Clostridium histolyticum* (Sigma-Aldrich, Dorset, UK). Islets were cultured for 48 h, and then subjected to an insulin-release studies as described previously [11]. The supernatants were stored at −20 °C for insulin radioimmunoassay, and the islets were retrieved to measure islet insulin content following acid-ethanol extraction [25]. To elucidate secretory pathways triggered by *A. squamosa*, these studies included evaluation of effects of known modulators of insulin secretion including diazoxide (K_ATP_-channel opener), tolbutamide (sulphonylurea and K_ATP_-channel blocker), verapamil (voltage-dependent Ca^2+^ channel blocker), IBMX (phosphodiesterase inhibitor), 30 mM KCl and 10 mM alanine which both depolarize the plasma membrane and induce Ca^2+^ influx. The amino acid alanine achieves this largely by co-transport with Na^+^ plus metabolism with ATP generation [26].

### 2.3. Membrane Potential and Intracellular Calcium ([Ca^2+^]_i_) Concentration

Changes in membrane potential and intracellular [Ca^2+^]i concentrations of BRIN-BD11 cells in response to exposure to hot water extract of *A. squamosa* were determined using a FLIPR Membrane Potential Assay Kit and a FLIPR Calcium 5 Assay Kit (Molecular Devices, Sunnyvale, CA, USA) as described previously [27]. A depolarising concentration of 30 mM KCl and 10 mM alanine were used as positive controls.

### 2.4. Glucose Uptake Assay

The 3T3L1 fibroblast cell monolayers were differentiated in culture to adipocytes as reported previously [28]. 50 µL of 200 µg/mL reconstituted plant extract with or without 100 nM insulin was added to each well of 3T3-L1 differentiated cells and incubated for 30 min at 37 °C in an atmosphere of 5% CO_2_ and 95% air. 2-NBDG, 2-(N-(7-Nitrobenz-2-oxa-1,3-diazol-4-yl) Amino)-2-Deoxyglucose (50 nM) was added. After 5 min, the wells were washed with ice-cold PBS and three to four coverslips were mounted in each slide and blocked with nail polish. Four images were taken of the four corners of the coverslips using a 10× magnification on the microscope. The fluorescence intensity was measured.

### 2.5. Glycation of Insulin

The effects of plant extract on in vitro protein glycation were determined as described previously [29]. Plant extract and glucose solutions were prepared in 10 mM sodium phosphate buffer (pH 7.4). 246.5 mM D-glucose and NaBH_3_CN (0.0853 gm/mL) were incubated with or without plant extract (50, 100, 200 μg/mL) in the presence of human insulin (1 mg/mL) at 37 °C for 24 h in a total volume 1 mL (pH 7.4). The reaction was stopped by addition of 30 μL of 0.5 M acetic acid. Separation and quantification of glycated and non-glycated insulin were performed using reversed-phase high-performance liquid chromatography [30]. Aminoguanidine, an established inhibitor of protein glycation was used as a positive control.

### 2.6. DPP-IV Enzyme Activity In Vitro

Effects of *A. squamosa* on DPP-IV enzyme activity were determined using a fluorometric method. Enzyme activity were determined using 96-well black-walled, clear-bottomed microplates (Greiner) containing 8 mU/mL of DPP-IV enzyme and 200 µM of substrate (Gly-Pro-AMC) as described previously [31]. Flex Station 3 (Molecular Devices, Sunnyvale, CA, USA) was used to measure the changes in fluorescence with an excitation and emission at 370 and 440 nm with 2.5 nm slit width. Established DPP-IV inhibitors, sitagliptin and vildagliptin were used as positive controls.

### 2.7. Starch Digestion

The starch digestion studies were performed as described previously [32]. Starch (100 mg) (Sigma-Aldrich, St. Louis, MO, USA) was dissolved in 3 mL of distilled water with or without plant extract (62.5–1000 µg/mL) and acarbose (62.5–1000 µg/mL), an established α-glucosidase inhibitor, as a positive control. 40 µl of 0.01% heat stable α-amylase (from Bacillus leicheniformis, Sigma-Aldrich, St. Louis, MO, USA) was added. After incubation at 80 °C for 20 min, the mixture was diluted to 10 mL and 1 mL was incubated with 2 mL of 0.1 M sodium acetate buffer (pH 4.75) and 30 µL of 0.1% amyloglucosidase from Rhizopus mold (Sigma-Aldrich, St. Louis, MO, USA) for 30 min at 60 °C. The samples were analysed for glucose liberation using liquid GOD/PAP (glucose oxidase/four-aminophenazone-phenol) method (Randox GL 2623). The samples were measured at 500 nm against a blank reagent at 37 °C.

### 2.8. Glucose Diffusion In Vitro

The in vitro glucose diffusion model was based on a cellulose ester (CE) dialysis tubes (20 cm × 7.5 mm, Spectra/Por^®^CE layer, MWCO: 2000, Spectrum, Amsterdam, The Netherland). Briefly, a 2 mL volume of 0.9% NaCl (BDH Chemicals Ltd., Poole, UK) supplemented with 220 mM glucose was placed into dialysis tube either in the presence or absence of plant extract/gaur gum (0.2–25 mg/mL). The latter agent is well known to increase viscosity and slow intestinal glucose absorption. Both ends of each tube were sealed tightly and placed in a 50 mL centrifuge tube (Orange Scientific, Orange, CA, USA) containing 0.9% NaCl (45 mL). The tubes were put on an orbital shaker and maintained at 37 °C during shaking for 24 h with samples taken for glucose analysis as described previously [33].

### 2.9. Animals

Sprague-Dawley male rats (Envigo UK, approximately 350–400 g) were fed a high-fat diet (20% protein, 45% fat and 35% carbohydrate; 26.15 KJ/g total energy percent (Special Diet Service, Essex, UK) 5–6 weeks before the start of experimentations. Age-matched rats maintained on standard rodent maintenance diet (10% fat, 30% protein and 60% carbohydrate making 12.99 KJ/g total energy, Trouw Nutrition, Cheshire, UK) were used as controls. All studies were approved by the Animal Welfare and Ethical Review Board (AWERB) at Ulster University and conducted in accordance to UK Animals (Scientific Procedures) Act 1986 and EU Directive 2010/63EU. All necessary steps were taken to prevent any potential animal suffering.

### 2.10. Oral Glucose Tolerance

To evaluate the effects of hot water extract of *A. squamosa* leaves and their isolated compound (rutin) on glycaemic control, oral glucose tolerance tests were performed using high-fat-fed rats (380–400 gm body wt.). Blood samples were collected at times indicated in Figures before and after oral administration of glucose (18 mmol/kg body weight) without and with plant extract (250 mg/5 mL/kg). Plasma was separated from the whole blood by centrifugation for 5 min at 12,000 rpm at 4 °C and stored at −20 °C prior to analysis of insulin. An Ascencia Contour glucose meter (Bayer, Newbury, UK) was used to measure blood glucose and insulin was determined by dextran-charcoal radioimmunoassay [11].

### 2.11. DPP-IV Enzyme Activity In Vivo

The effects of plant extract on activity of DPP-IV was determined in plasma by a fluorometric assay based on the liberation of AMC (7-Amino-4-Methyl-Coumarin) from DPP-IV substrate, Gly-Pro-AMC with the modification as described previously [34]. Blood samples were obtained from high-fat-fed rats at times indicated in Figure after oral administration of plant extract (250 mg/5 mL/kg), the established DPP-IV inhibitors sitagliptin (10 µmol/5 mL/kg) and vildagliptin (10 µmol/5 mL/kg) or saline control.

### 2.12. Glucose Homeostasis after 9-Days Treatment with Hot Water Extract of A. squamosa Leaves in High-Fat Fed Rats

High-fat fed adult, male Sprague-Dawley rats were administered twice-daily oral gavage dose of either saline vehicle (0.9% *w*/*v*) or plant extract (250 mg/5 mL/kg body weight) for 9 consecutive days. Food intake, fluid consumption, body weight and circulating glucose and insulin were measured before and at regular intervals. Glucose tolerance (18 mmol/kg body weight) was evaluated after 6 days of treatment. At the end of study, pancreata were collected for analysis of islet morphology and pancreatic insulin content [11].

### 2.13. Islet Morphology after 9-Days Treatment with Hot Water Extract of A. squamosa Leaves in High-Fat Fed Rats

Pancreata excised from high-fat fed (treated or untreated) and normal rats (control) were placed in 4% paraformaldehyde at 4 °C for 48 h and an automated tissue processor was used to process the tissues. Tissue staining and their analysis were conducted as previously described [35]. Sections (5 to 8 μM) were placed on slides and dewaxed and rehydrated. A 2% BSA buffer was used for 30 min for blocking, and slides were incubated overnight at 4 °C with primary antibody (mouse anti-insulin (1:500) and guinea pig anti-glucagon (1:400). The slides were incubated at room temperature with secondary antibody mixture (Alexa Fluor 594 goat anti-mouse antibody and Alexa Fluor 488 goat anti-guinea pig antibody). After staining the nucleus with 4′,6-diamidino-2-phenylindole (DAPI), the slides were mounted using anti-fade mounting medium. Then the slides were subjected for analysis using tetramethylrhodamine isothiocyanate (594 nm) or fluorescein isothiocyanate filters (488 nm) using a fluorescent microscope (Olympus System Microscope BX51, Olympus Instruments, London, UK). Images were captured using a DP70camera adapter system. Cell^F imaging software (Olympus System Microscope BX51, Olympus instruments, London, UK) was used to determine islet distribution, islet area, beta cell and alpha cell area.

### 2.14. Purification of Crude Extracts

Crude extracts were re-dissolved in solvent (0.12% (*v/v*) TFA/Water) and purified by reversed-phase high-performance liquid chromatography. The extract solution was injected onto a (22 × 250 mm) Vydac 218TP1022 10 μm (C-18) reversed-phase HPLC column (Grace, Deerfield, IL, USA) equilibrated with 0.12% (*v/v*) TFA/water at a flow rate of 5 mL/min. The concentration of acetonitrile within the eluting solvent was expanded using linear gradients to 20% over 10 min, 70% over a period of 40 min. The wavelength of 254 nm and 360 nm were used to measure the absorbance and fractions were collected according to the appearance of peaks in individual time points. Further, insulinotropic fractions were purified using Vydac 208TP510 (10 × 250 mm) semi-preparative stainless steel 5 μm C-18 column (Phenomenex, London, UK). Acetonitrile concentration in the eluting solvent was same as above at a flow rate of 1 mL/min.

### 2.15. Structural Characterization of Purified Extracts

Molecular weights of fractions (peak samples) were determined using Liquid Chromatography-Mass Spectrometry (LC-MS) via Electrospray ionization mass spectrometry (ESI-MS). Fractions (peak samples) were separated on a Spectra System LC (Thermo Separation Products) using a Kinetex 5 µm F5 LC column ((150 × 4.6 mm) (Phenomenex)). A 10 µL volume was chromatographed at ambient temperature using gradient elution. The column was allowed to equilibrate for 10 min at 80% water: 20% acetonitrile, then increasing to 70% acetonitrile over 20 min and returning to the initial conditions for 5 min to allow re-equilibration before the next injection. A flow rate of 1 mL/min was maintained over the 35 min run time with UV detection at 220–360 nm. The LC effluent was routed to an LCQ electrospray ion trap mass spectrometer (Thermo Finnigan, San Jose, CA, USA) for mass characterization. The system was controlled using Xcalibur software (Thermo Scientific, CA, USA) and operated in negative ion mode, scanning over the mass range *m/z* 150.0–1200.0 Da. Nitrogen gas for the sheath and auxiliary gas were set 65 and 8 arbitrary units respectively. The spray voltage was set to 4.5 kV and the heated capillary temperature maintained at 250 °C. The precision level of the instrument was ±0.02%.

### 2.16. Confirmation of Extract Purity and Identity

All nuclear magnetic resonance (NMR) spectra were physically staged and routinely baseline corrected as described previously by Grace et al. [36] for compound identity confirmation followed by HPLC and LC-MS analysis. NMR spectra had been recorded on a 600 MHz Bruker AVIII HD (Bruker BioSpin, Coventry, UK) spectrometer outfitted with a 5 mm BBO H & F cryogenic test. Standard one-dimensional composite pulse sequencing (zgcppr) was used to obtain ^1^H NMR spectra. The ^13^C NMR spectra were obtained with the aid of the use of the reverse gated-decoupling pulse sequence (zgig) and the purchase parameters were set as described previously [36].

### 2.17. Statistical Analysis

A statistical analysis software for windows, Graph Pad prism 5, used for all types of analysis and interpretation of data. All data were analysed by unpaired Student’s t test (nonparametric, with two-tailed *p*-values) and one-way ANOVA with Bonferroni post hoc tests wherever applicable. To plot area under the curve (AUC) with baseline correction, trapezoidal rules were followed. Values were presented as mean ± SEM; the significant limit was determined as *p* < 0.05.

## 3. Results

### 3.1. Effects of A. squamosa Leaves on Insulin Release from BRIN-BD11 Cells

**Figure 1:**
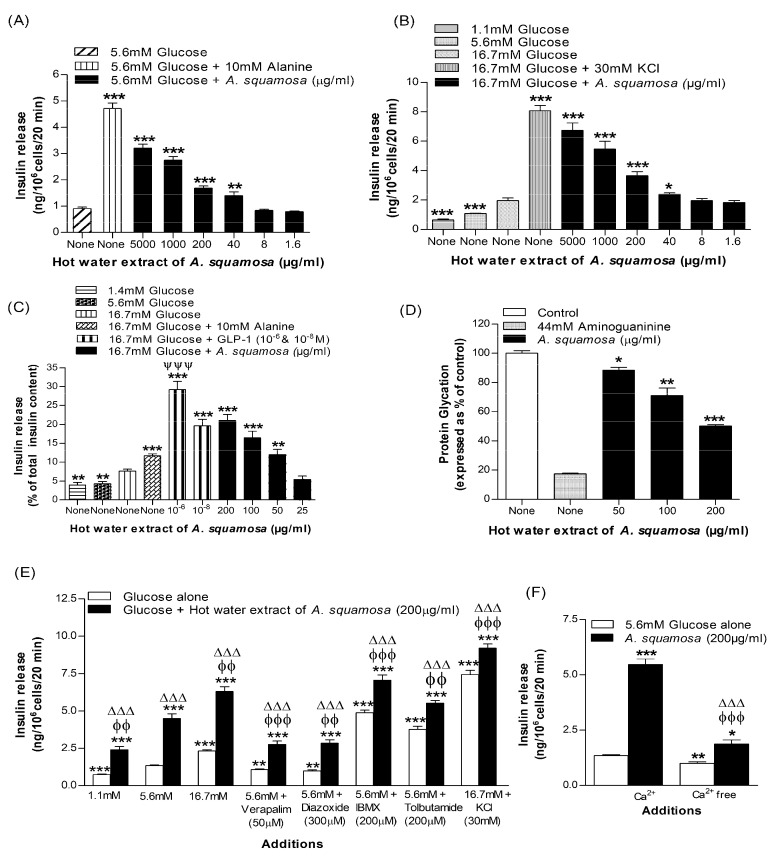
Dose-dependent effects hot water extract of *A. squamosa* leaves on insulin release from (**A**,**B**) BRIN-BD11 cells and (**C**) isolated pancreatic islets, (**D**) protein glycation, (**E**) insulin secretion in the presence or absence of established stimulators or inhibitors and (**F**) extracellular calcium from BRIN BD11 cells. Values are Mean ± SEM for n = 8 and 4 for insulin release and n = 3 for protein glycation. * *p* < 0.05, ** *p* < 0.01 and *** *p* < 0.001 compared to control (5.6/16.7 mM glucose and 220 mM glucose + insulin (1 mg/mL)). ^ϕ^ *p* < 0.05, ^ϕϕ^ *p* < 0.01 and ^ϕϕϕ^ *p* < 0.001 compared to 5.6 mM glucose in the presence of the extract for (E,F). ^Δ^ *p* < 0.05, ^ΔΔ^ *p* < 0.01, ^ΔΔΔ^ *p* < 0.001 compared to respective incubation in the absence of the extracts for (**E**,**F**). ^ΨΨΨ^ *p* < 0.001 compared to alanine in the presence of 16.7 mM glucose for (**C**).

Insulin secretion from BRIN-BD11 cells was increased (*p* < 0.05–*p* < 0.001) in a concentration (40–5000 μg/mL) dependent manner by hot water extract of *A. squamosa* at both 5.6 mM and 16.7 mM glucose (Figure 1A,B). Insulin secretagogues, alanine (10 mM) and membrane depolarizing agent, KCl (30 mM) were used as positive controls. Extract concentrations up to 200 μg/mL did not affect cell viability, but higher concentrations increased release of LDH levels by 16% to 60% (data not shown).

### 3.2. Effects of A. squamosa Leaves on Insulin Release from Isolated Mouse Islets

Hot water extract of *A. squamosa* induced concentration dependent (25–200 μg/mL) stimulation of glucose-induced insulin secretion by 1.4–2.5-fold compared to control (16.7 mM glucose alone) (*p* < 0.01–0.001, Figure 1C). Glucose dependent insulin secretion modulator, GLP-1 (10^−6^ and 10^−8^ M) and alanine (10 mM) used as positive controls also significantly increased insulin release (*p* < 0.001; Figure 1C).

### 3.3. Effects of A. squamosa Leaves on Protein Glycation In Vitro

The effects of hot water extract of *A. squamosa* (HWAS) on protein glycation are illustrated in Figure 1D. Aminoguanidine (44 mM) used as a positive control inhibited insulin glycation by 82.7% (*p* < 0.001, Figure 1D). Insulin glycation was decreased by 12–50% in a concentration-dependent manner by the 50–200 µg/mL hot water extract (*p* < 0.05–0.001, Figure 1D).

### 3.4. Effects of A. squamosa Leaves on Insulin Release in the Presence of Known Modulators of Insulin Secretion and Absence of Extracellular Calcium from BRIN BD11 Cells

Additional studies were performed to understand the mechanism underlying the insulin secretory actions of non-toxic concentration (200 µg/mL) of *A. squamosa* extract. As shown in Figure 1E, *A. squamosa* induced insulin release (*p* < 0.001) was enhanced by various modulators including, 16.7 mM glucose (*p* < 0.05), isobutylmethylxanthine (IBMX; *p* < 0.001) and tolbutamide (*p* < 0.001). Cyclic AMP production and insulin secretion were amplified using the phosphodiesterase inhibitor IBMX and the sulphonylurea, tolbutamide. On the other hand, the presence of diazoxide, a K_ATP_ channel opener and verapamil, voltage-gated calcium channel blocker significantly inhibited the insulin releasing effect by decreasing the influx of intracellular Ca^2+^ (*p* < 0.001). The extract maintained its ability to increase insulin secretion from the cells depolarized with 30 mM KCl (*p* < 0.001; Figure 1E) but absence of added Ca^2+^ ions to the incubation buffer reduced but did not entirely abolish the insulin-releasing activity of the plant (*p* < 0.001; Figure 1F).

### 3.5. Effects of A. squamosa Leaves on Membrane Depolarization and Intracellular Calcium from BRIN BD11 Cells

**Figure 2:**
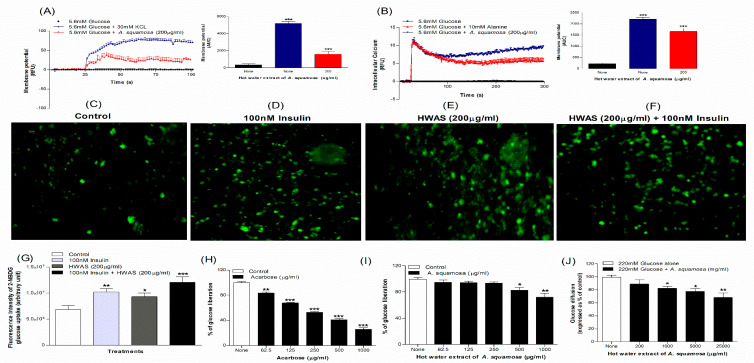
Effects of hot water extract of *A. squamosa* leaves on (**A**) membrane potential and (**B**) intracellular calcium in BRIN BD11 cells, (**C**–**G**) glucose uptake in differentiated 3T3L1 adipocyte cells, (**H**,**I**) starch digestion and (**J**) glucose diffusion in vitro. Changes of fluorescence intensity in differentiated 3T3L1 adipocytes incubated with hot water extract of *A. squamosa* leaves (HWAS; 200 µg/mL) + 50 nM 2-NBDG in the (**E**) absence or (**F**) presence of 100 nM insulin. Images were taken at 10× magnification. Values are Mean ± SEM for n = 6 for membrane potential and intracellular calcium, n = 4 for glucose uptake, starch digestion and glucose diffusion. * *p* < 0.05, ** *p* < 0.01 and *** *p* < 0.001 compared to control.

As shown in Figure 2A,B, the hot water extract induced membrane depolarization (*p* < 0.001, Figure 2A) and caused a significant increase in intracellular calcium ion concentration in BRIN-BD11 cells at 5.6 mM glucose (*p* < 0.001, Figure 2B). Both, KCl (30 mM) and alanine (10 mM) were used as positive controls caused depolarization and increased intracellular calcium ions (*p* < 0.001, Figure 2A,B) compared to the control (5.6 mM glucose alone).

### 3.6. Effects of A. squamosa Leaves on Glucose Uptake and Insulin Action

The effects of *A. squamosa* on glucose uptake and insulin action were determined by 2-NBDG (2-(N-(7-Nitrobenz-2-oxa-1,3-diazol-4-yl) Amino)-2-Deoxyglucose) fluorescent hexose, a glucose analogue using the 3T3L1 adipocyte cells. Figure 2C–F show the microscopic fluorescence intensity images of 2-NBDG uptake. *A. squamosa* (200 μg/mL) increased glucose uptake by 1.2-fold in 3T3L1 differentiated adipocyte cells compared to control (*p* < 0.05–0.001, Figure 2G). This effect was not associated with toxic effects on 3T3L1 cells. In the presence of insulin (100 nM), hot water extract of *A. squamosa* leaves showed the greatest cellular intensity of 2-NDBG fluorescence (*p* < 0.001, Figure 2G). Hot water extract increased glucose uptake in 3T3L1 adipocyte cells by 30% in the presence of insulin (100 nM) and by 20% in the absence of insulin (Figure 2G).

### 3.7. Effects of A. squamosa Leaves on Starch Digestion In Vitro

The effects of *A. squamosa* on starch digestion are presented in Figure 2H,I. Acarbose (62.5–1000 µg/mL) used as positive control produced 17–72% concentration-dependent decrease in starch digestion (Figure 2H). At the higher concentrations (500–1000 µg/mL), the hot water extract of *A. squamosa* significantly (*p* < 0.05–0.01, Figure 2I) inhibited starch digestion by 18–28%.

### 3.8. Effects of A. squamosa Leaves on Glucose Diffusion In Vitro

Hot water extract of *A. squamosa* resulted in a significant (*p* < 0.05–0.01) inhibition of glucose diffusion over 24-h incubation as compared to the control. As shown in Figure 2J, the extract decreased the total diffusion of glucose by 10–30% with maximal effect at a concentration of 25,000 µg/mL (30%, Figure 2J).

### 3.9. Effects of A. squamosa Leaves on DPP-IV Enzyme Activity In Vitro

**Figure 3:**
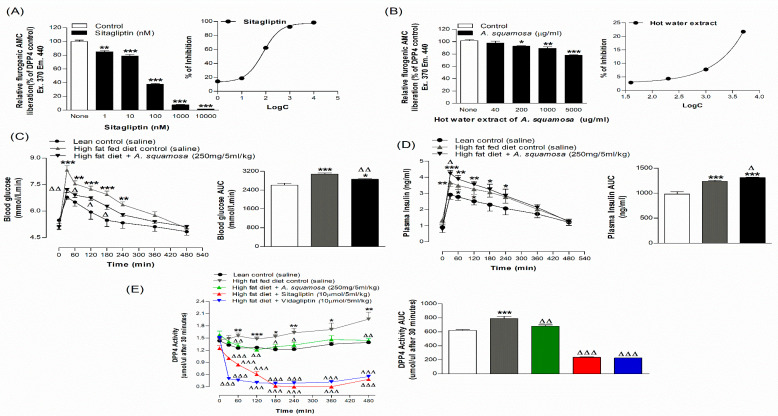
Effects of hot water extract of *A. squamosa* on (**A**,**B**) DPP-IV enzyme activity in vitro, (**C**,**D**) glucose tolerance and (**E**) plasma DPP-IV in high-fat fed rats fasted for 6 h. Glucose tolerance, plasma insulin and DPP-IV enzyme activity were measured prior to and after oral administration of glucose alone (18 mmol/kg body weight, control) or with *A. squamosa* leaves (250 mg/5 mL/kg body weight). Values are Mean ± SEM for n = 3 for DPP-IV enzyme activity in vitro and n = 6 for glucose tolerance, plasma insulin and DPP-IV in vivo. * *p* < 0.05, ** *p* < 0.01 and *** *p* < 0.001, compared to normal control and ^Δ^ *p* < 0.05, ^ΔΔ^ *p* < 0.01 and ^ΔΔΔ^ *p* < 0.01 compared to high-fat fed diet control.

The in vitro DPP-IV enzyme inhibitory effects of sitagliptin and hot water extract of *A. squamosa* were evaluated as shown in the Figure 3A,B. Sitagliptin an established inhibitor of DPP-IV enzyme, reduced enzyme activity in a dose-dependent manner (1–10 µM) by 15–98% (*p* < 0.01–0.001, Figure 3A). Hot water extract significantly inhibited DPP-IV activity by 8–22% at concentrations between 200 and 5000 µg/mL (*p* < 0.05–0.001; Figure 3B).

### 3.10. Effects of A. squamosa Leaves on Oral Glucose Tolerance and Insulin Responses

Oral administration of hot water extract of *A. squamosa* (250 mg/5 mL/kg) with glucose (18 mmol/kg body weight) significantly (*p* < 0.05–0.01) improved glucose tolerance in high-fat fed rats compared to glucose alone (Figure 3C). This was associated with a significantly increased plasma insulin response at 30 min (*p* < 0.05, Figure 3D). It is notable that high-fat fed rats had a significantly higher level of blood glucose (*p* < 0.01–0.001) and plasma insulin (*p* < 0.05–0.01) compared to the normal lean rats (Figure 3C,D).

### 3.11. Effects of A. squamosa Leaves on DPP-IV Enzyme Activity In Vivo

High-fat fed rats receiving oral glucose (18 mmol/kg) plus extract (250 mg/5 mL/kg) showed a reduction in DPP-IV enzyme activity (*p* < 0.05–0.01, Figure 3E) from 30 min onwards with 20–24% decrease compared to the high-fat fed control rats (glucose alone). Sitagliptin and vidagliptin (10 µmol/5 mL/kg), established inhibitors of DPP-IV used as positive controls, provoked reduced enzyme activity by 69–71% from 30 min (*p* < 0.001, Figure 3E). 

### 3.12. Effects of A. squamosa Leaves on Food Intake, Energy Intake, Fluid Intake, Body Weight, Non-Fasting Blood Glucose, Plasma Insulin and DPP-IV Concentration in High-Fat Fed Rats

**Figure 4:**
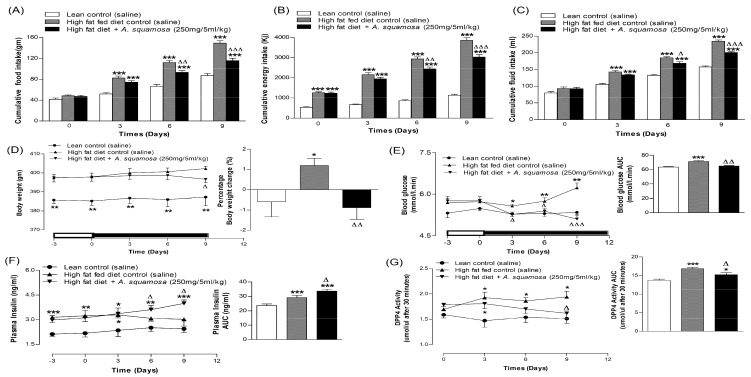
Effects of 9 days treatment of twice daily oral administration of hot water extract of *A. squamosa* leaves on (**A**) food intake, (**B**) energy intake, (**C**) fluid intake, (**D**) body weight, (**E**) blood glucose, (**F**) plasma insulin (**G**) and DPP-IV enzyme activity in high-fat fed rats. *A. squamosa* leaves extract was administered orally (250 mg/5 mL/kg body weight) twice daily. Values are Mean ± SEM for n = 8 rats. * *p* < 0.05, ** *p* < 0.01 and *** *p* < 0.001 compared to lean control. ^Δ^ *p* < 0.05, ^ΔΔ^ *p* < 0.01 ^ΔΔΔ^ *p* < 0.001 compared to high-fat fed diet control at corresponding time-point.

**Figure 5:**
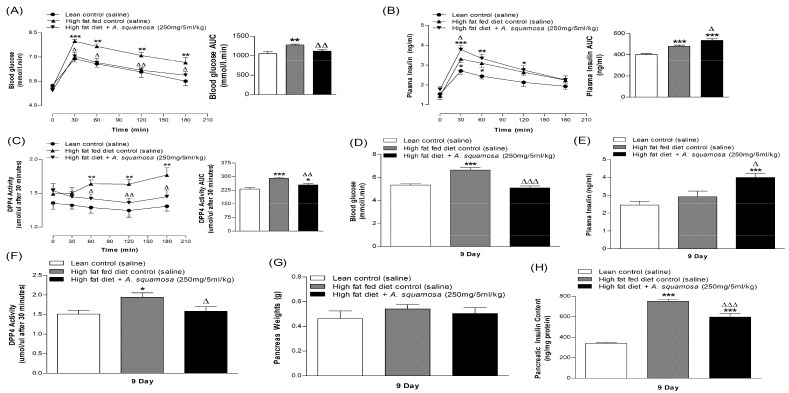
Chronic effects of twice daily oral administration of hot water extract of *A. squamosa* leaves on (**A**) glucose tolerance (**B**) plasma insulin and (**C**) plasma DPP-IV on day 6 and (**D**) blood glucose, (**E**) plasma insulin, (**F**) plasma DPP-IV, (**G**) pancreas weight and (**H**) pancreatic insulin content on day 9 in high-fat fed rats. *A. squamosa* leaves extract was administered orally (250 mg/5 mL/kg body weight) twice daily. Glucose tolerance was assessed by oral administration of glucose (18 mmol/kg). Values are Mean ± SEM with n = 8. * *p* < 0.05, ** *p* < 0.01 and *** *p* < 0.001 compared to lean control. ^Δ^ *p* < 0.05, ^ΔΔ^ *p* < 0.01 ^ΔΔΔ^ *p* < 0.001 compared to high-fat fed diet control at corresponding time-point.

Administration of leaf extract (250 mg/5 mL/kg) to high-fat fed rats orally twice daily for 9 days reduced food intake, energy intake, fluid intake and blood glucose by 16%, 17%, 10% and 7% respectively, from day 6 onwards (*p* < 0.05–0.001, Figure 4A–C,E and Figure 5D). This coincided with reduction in the body weight with a 16% change from starting values (*p* < 0.05–0.01; Figure 4D). The extract also enhanced the plasma insulin levels from 6-days onwards compared to high-fat fed diet controls (*p* < 0.05; Figure 4F and Figure 5E). This was accompanied by 16% decrease of DPP-IV enzyme activity compared to high-fat fed diet control alone (*p* < 0.05, Figure 4G and Figure 5F).

### 3.13. Effects of A. squamosa Leaves on Glucose Tolerance and, Associated Plasma Insulin and DPP-IV Levels in High-Fat Fed Rats

After 6 days oral administration of *A. squamosa* (250 mg/5 mL/kg), oral glucose tolerance (18 mmol/kg, body weight) was significantly improved (*p* < 0.001) from 30 min onwards compared to high-fat fed controls (*p* < 0.05–0.01, Figure 5A). Similarly, *A. squamosa* treatment was associated with a sharp increase in insulin secretion by 30 min after the oral glucose challenge (Figure 5B), with 1.4-fold greater insulin responses (*p* < 0.05) than high-fat fed controls. *A. squamosa* (250 mg/5 mL/kg) also reduced DPP-IV enzyme activity by 13–17% on 6-days treatment from 60 min onwards during oral glucose tolerance test (Figure 5C; (*p* < 0.05–0.01)).

### 3.14. Effects of A. squamosa Leaves on Pancreatic Insulin and Islet Morphology in High-Fat Fed Rats

**Figure 6:**
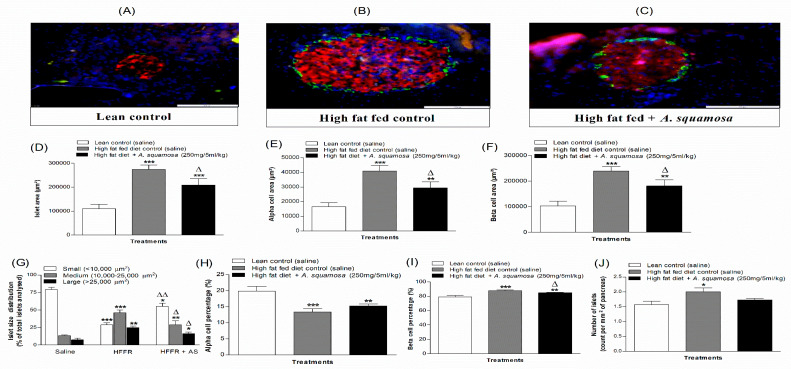
Effects of 9 days treatment of hot water extract of *A. squamosa* leaves on islet morphology in high-fat fed rats. Representative images of (**A**) lean control, (**B**) high-fat fed control and (**C**) high-fat fed rats treated with hot water extract of *A. squamosa* (250 mg/5 mL/kg), showing insulin in red, glucagon in green and DAPI in blue, (**D**) islet area, (**E**) alpha cell area, (**F**) beta cell area, (**G**) islet size distribution, (**H**) alpha cell percentage, (**I**) beta cell percentage and (**J**) number of islets (per mm^2^) respectively. Values are mean ± SEM for n = 8 (~100 islets per group). * *p* < 0.05, ** *p* < 0.01 and *** *p* < 0.001 compared to lean control. ^Δ^ *p* < 0.05 and ^ΔΔ^ *p* < 0.01 compared to high-fat fed diet alone (control).

Pancreatic weights were similar in all groups of rats (Figure 5G) but pancreatic insulin content was increased by 43% in high-fat fed treated rats compared to lean controls (*p* < 0.001, Figure 5H). Treatment with *A. squamosa* significantly reduced the elevated insulin content by 21% (*p* < 0.001, Figure 5H). Pancreatic islet morphology was evaluated in (A) lean-control, (B) high-fat fed diet control and (C) high-fat fed diet rats treated for 9 days with hot water extract of *A. squamosa* (Figure 6A–J)). In the high-fat fed group, the number of islets per mm^2^ was greater than in the lean control group (*p* < 0.05, Figure 6J). Beta and alpha cell areas were also increased significantly as compared to lean controls (*p* < 0.01–0.001, Figure 6E,F). *A. squamosa* produced a significant 15% reduction of the islet area (*p* < 0.05, Figure 6D). The number of large and medium size islets was reduced significantly (*p* < 0.05) and the number of small size islets increased (*p* < 0.01) after *A. squamosa* treatment (Figure 6G). The beta and alpha cell areas were also decreased markedly (*p* < 0.05) in the treated group (Figure 6E,F). Notably, the proportion of beta cells was higher in high-fat fed groups compared to the normal rats and the % of alpha cells was decreased (*p* < 0.01–0.001, Figure 6H,I). Furthermore, the proportion of beta cells were decreased in the treated group compared to high-fat fed control rats (*p* < 0.05, Figure 6I).

### 3.15. Purification and Structural Characterization of Purified Extract

**Figure 7:**
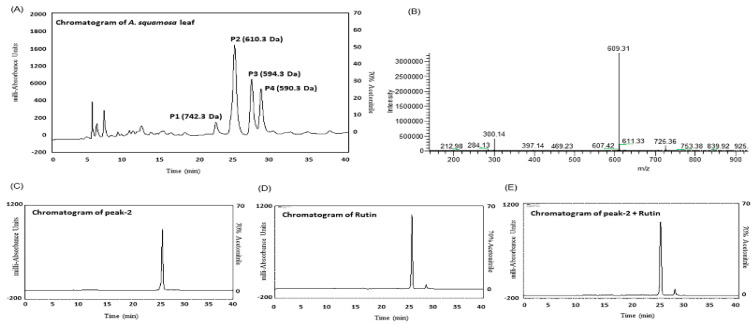
Representative HPLC profile of (**A**) hot water extract of *A. squamosa* leaves, (**B**) LC-MS analysis of peak samples were separated on a Spectra System LC using a Kinetex 5 µm F5 LC column ((150 × 4.6 mm) (Phenomenex), (**C**) Peak 2, (**D**) Rutin and (**E**) peak 2 + Rutin eluting from analytical C-18 column for over 40 min. The column was equilibrated with 0.12% (*v/v*) TFA/water at flow rate of 5.0 and 1.0 mL/min respectively. The concentration of the eluting solution was raised using linear gradients from 0% to 20% acetonitrile over 10 min, to 70% over 40 min. Details of peaks corresponding to *A. squamosa* leaves and rutin are presented in the chromatogram. UV detection was set at 220 and 360 nm and 1 mg/mL sample was injected for each run. In the chromatogram, P represents the peak in different retention time (RT). The mass-to-charge ratio (*m/z*) versus peak intensity was determined. Fragments of “peak-1 to 4” represents the molecular weight of unknown compounds with *m/z* 742.3 to 590.3 Da (data not shown) respectively.

**Figure 8:**
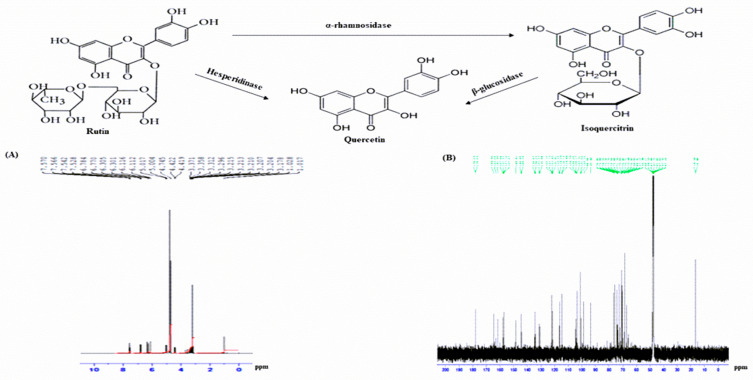
(**A**) ^1^H-NMR, (**B**) C^13^-NMR spectrum and isolated compound (rutin) from peak-2 sample obtained following RP-HPLC of hot water extract of *A. squamosa* leaves and its metabolites, isoquercitrin and quercetin. Proton-decoupled natural abundance C^13^-NMR and ^1^H-NMR spectrum of peak-2 sample of hot water extract of *A. squamosa* leaves (obtained from chromatograph over the period of 0 to 70% acetonitrile from 0 to 40 min with retention time of 25 min) at 40 °C. The spectrum was obtained at 600 and 150 MHz after 119,044 transients (14 h) by the pulsed Fourier transform method on a Varian XL-100 A spectrometer. Representative structure of flavonoids, corresponding to the structure of rutin (Quercetin 3-rutinoside), isoquercitrin and quercetin are C_27_H_30_O_16_), C_21_H_20_O_12_ and C_15_H_10_O_7_.

Extract was purified using RP-HPLC and peak fractions were detected using UV detector of 254 and 360 nm and mass to charge ratio of peak samples were determined using LCMS (Figure 7A–E). Molecular weights of peaks 1, 3 and 4 were 742.3, 594.3 and 590.3 Da (data not shown) whereas the molecular weight of peak 2 was 610.3 Da (Figure 7B). Figure 7C–E shows an identical retention time for (C) peak-2, (D) rutin and (E) peak-2 + rutin. Further structural characterization of the compounds was obtained by NMR and the characteristics of the isolated compounds depicted in Figure 8A for ^1^H and Figure 8B for ^13^C NMR are as presented below.

Rutin (C_27_ H_30_ O_16_): EI-MS *m/z* 610.3 Da [M]; (600 MHz, CD_3_OD, ^1^H-NMR (δ in ppm): 7.57 (dd, 1H, *J* = 2.4 Hz, *J* = 8.4 Hz), 6.78 (d, 1H, *J* = 8.4 Hz), 6.30 (s, 1H), 6.11 (s, 1H), 5.01 (s, 1H), 4.74 (d, 1H, *J* = 7.8 Hz), 4.2 (d, 1H, *J* = 1.8 Hz), 3.37–3.21 (m, 7H), 3.20 (d, 2H, *J* = 17.4 Hz), 1.18 (d, 3H, *J* = 6.6 Hz). ^13^C NMR (150 MHz, CD_3_OD, δ in ppm); 178.2 (CO-9), 164.6 (C-13), 161.8 (C-11), 157.9 (C-15), 157.0 (C-7), 148.8 (C-3), 144.4 (C-4), 134.4 (C-8), 122.1 (C-6), 121.7 (CH-1), 116.5 (CH-2), 114.7 (CH-5), 104.5 (C-10), 98.5 (CH-12), 93.4 (CH-14), 101.0 (C1-G), 74.3 (C2-G), 75.8 (C3-G), 72.5 (C4-G), 76.8 (C5-G), 68.3 (C6-G), 103.3 (C1-R), 73.7 (C2-R), 72.4 (C3-R), 72.5 (C4-R), 73.9 (C5-R), 16.5 (C6-R) (the letters R and G respectively, symbolise the signals of glucose and rhamnose molecules).

For the primary flavonoid, rutin, the HPLC conditions outlined in the experimental section were used to separate and quantify rutin within the samples. This revealed that amount of rutin in 1 mg/mL of *A. squamosa* leaves extract after purification was 15–20%. This is in strong agreement with previous studies the identification of the compound confirmed by HPLC, LCMS and NMR and their derivatives: isoquercitrin and quercetin are presented in Figure 8.

### 3.16. Effects of Peak Samples of A. squamosa Leaves on Insulin Release In Vitro

**Figure 9:**
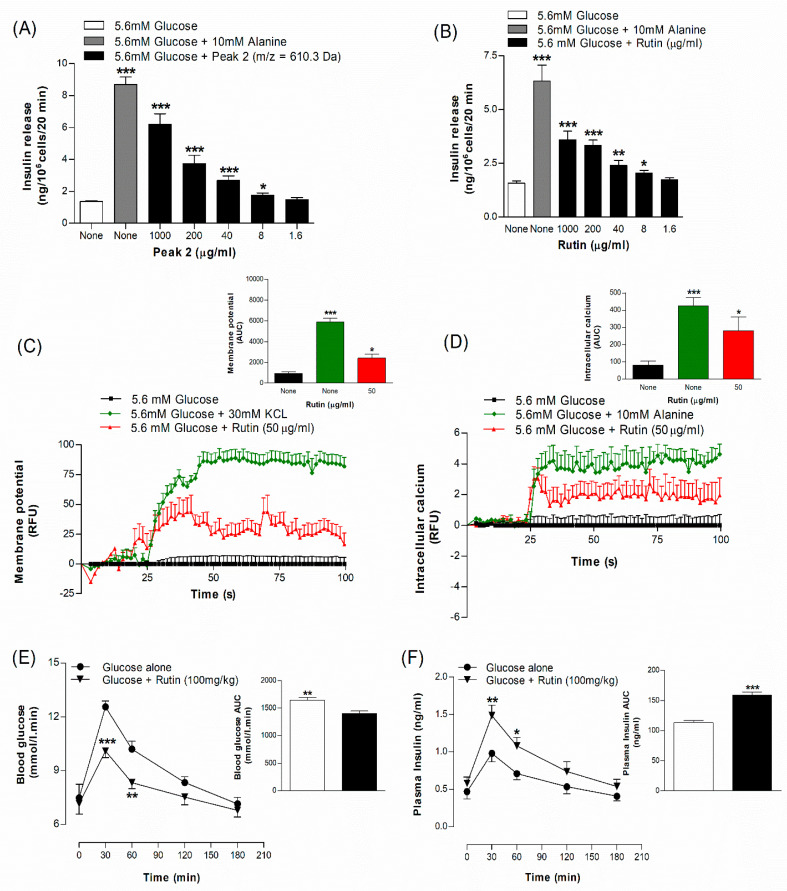
Dose-dependent effects of (**A**) peak 2 sample of hot water extract of *A. squamosa* leaves and (**B**) rutin on insulin release from BRIN-BD11 cells, (**C**) membrane potential and (**D**) intracellular calcium expressed as RFU and respective area under the curve, (**E**) glucose tolerance and (**F**) plasma insulin. Mice were fasted for 12 h and administered glucose (18 mmol/kg body weight) by oral gavage with or without rutin (100 mg/kg body weight). Values are Mean ± SEM for n = 8 for insulin release, n = 6 for membrane potential and intracellular calcium and n = 7 for glucose tolerance and plasma insulin. * *p* < 0.05, ** *p* < 0.01 and *** *p* < 0.001 compared to control.

The effects of a range of concentrations of peak sample of *A. squamosa* leaf on insulin secretion from BRIN-BD11 cells are illustrated in Figure 9A. In these studies, alanine (10 mM) was used as a positive control. There was a concentration-dependent release of insulin observed by the peak-2 fraction (610.3 Da, 8 to 1000 µg/mL)) compared to control (5.6 mM glucose) (*p* < 0.05–0.001, Figure 9A). Peak-3 and peak-4 stimulated insulin release only at higher concentrations (1000 µg/mL) compared to control (5.6 mM glucose) (data not shown). The cell viability was not affected by various concentrations of the peaks between 8 to 1000 µg/mL (data not shown).

### 3.17. Effects of Isolated Compound Rutin on Insulin Release In Vitro

The effects of a range of concentrations of rutin, isolated from *A. squamosa* leaves, on insulin secretion from BRIN-BD11 cells are illustrated in Figure 9B. In these studies, alanine (10 mM) was used as a positive control. Rutin, stimulated insulin secretion at concentration range 8–1000 µg/mL (*p* < 0.05–0.001, Figure 9B). LDH release was increased at a higher concentration of 1000 µg/mL (data not shown).

### 3.18. Effects of Isolated Compound Rutin on Beta Cell Membrane Depolarization and Intracellular Calcium Ion Concentration

Rutin (50 μg/mL) induced membrane depolarization in the BRIN-BD11 cells at 5.6 mM glucose (Figure 9C). The magnitude of the effect was lower than that generated by a high depolarizing 30 mM concentration of KCl but was considerably greater (*p* < 0.05) than that produced by glucose alone. Similarly, rutin (50 μg/mL) and alanine (10 mM) produced a significant increase in intracellular Ca^2+^ (*p* < 0.05; Figure 9D).

### 3.19. Effects of Isolated Compound Rutin on Oral Glucose Tolerance and Insulin Responses

Oral administration of rutin (100 mg/kg body weight) with glucose (18 mmol/kg) to mice significantly (*p* < 0.01–0.001) lowered blood glucose during oral glucose tolerance test at 30 and 60 min by 18–20% compared to the control (glucose alone) (Figure 9E). Rutin also increased plasma insulin at 30 and 60 min by 30–33% following administration with glucose (*p* < 0.05–0.01, Figure 9F).

## 4. Discussion

The *A. squamosa*, also known as “ata” in Bengali, has been claimed as a potential anti-diabetic plant [19,20]. However, its mechanism of action(s) is still poorly understood. In this study, we investigated a wide range of potential antidiabetic effects of *A. squamosa* hot water extract including in vitro studies on insulin secretion, cellular glucose uptake, DPP-IV activity, starch digestion, glucose diffusion and glycation. Moreover, we conducted in vivo studies in high-fat fed rats to evaluate acute and longer-term actions on glucose homeostasis, islet morphology and pancreatic insulin content.

Extract of *A. squamosa* leaves stimulated concentration-dependent insulin secretion from clonal BRIN BD11 cells as well as potentiating glucose-induced insulin release from isolated mouse islets. Mechanistic studies performed with fluorescent indicator dyes and modulators of insulin release, such as diazoxide (K_ATP_-channel opener) and verapamil (voltage-dependent Ca^2+^ channel blocker), to interrogate specific signal transduction pathways revealed an insulinotropic action dependent on membrane depolarization and influx of intracellular Ca^2+^. However, effects persisted Ca^2+^ free buffer and in the presence of the sulphonylurea, tolbutamide, or 30 mM KCl which both strongly depolarize beta cells. This suggests additional complementary actions of *A. squamosa* via K_ATP_ and Ca^2+^-independent pathways involving adenylate cyclase/cAMP, phosphatidylinositol kinases or direct impacts on exocytotic machinery [11]. Consistent with the former, effects of *A. squamosa* were significantly enhanced by cAMP phosphodiesterase inhibition using IBMX. Interestingly, traditional use of *A. squamosa* in asthma treatment [37] involves elevation of cAMP in bronchial smooth muscles, relaxing the airway passage and downregulating cellular replication in smooth muscles [38].

Using differentiated 3T3L1 adipocytes, *A. squamosa* was shown to enhance cellular glucose uptake in the absence but particularly in presence of sub-maximal stimulatory concentration of insulin. This strongly suggests that a component of the antidiabetic action of *A. squamosa* involves promotion of insulin action as well as insulin secretion. In target cells, insulin initiates a signal transduction via binding to insulin receptors which increases glucose uptake via recruitment of GLUT4 to plasma membrane with subsequent metabolism and storage [39]. Previous studies have shown that activation of Akt and p70 S6 kinase can increase glycogen, lipid, and protein synthesis and in turn promote glucose uptake [40]. However further studies are required to elucidate the possible involvement of this and other mechanisms of action of *A. squamosa* extract.

Additional antidiabetic actions of *A. squamosa* possibly involve effects in the GI-tract, so effects on carbohydrate digestion were explored in vitro. Acarbose, an α-glucosidase inhibitor, was used as a reference standard which almost abolished glucose liberation from starch. *A. squamosa* extract was also effective in inhibiting starch digestion which can be envisaged to greatly retard glucose absorption in vivo. Interestingly, previous studies on plant alkaloids and flavonoids revealed their ability to exert α-glucosidase inhibitory action [29,41] which are consistent with our present observations. We also used a simple in vitro system to study effects of *A. squamosa* extract on glucose diffusion [33]. Like our positive control, guar gum, *A. squamosa* substantially reduced glucose diffusion in this in vitro model, mediated primarily by viscosity changes.

Glycation is a common post translational modification of proteins in diabetes and is strongly implicated in the genesis of long-term complications [42]. Insulin glycation is an example of glycation of short-lived protein that can lead to insulin resistance because of its reduced biological activity [43]. In our study, *A. squamosa* inhibited the insulin glycation in vitro, suggesting its utility in helping to prevent glycation of functional and structural proteins in the progression of insulin resistance and diabetic complications. Recent studies of *A. squamosa* have also reported significant anti-oxidant properties [44] that might also protect against beta cell damage and apoptosis.

These in vitro observations are consistent with potential strong antidiabetic efficacy of *A. squamosa* and our studies were therefore extended to examine rats fed high-fat diet with obesity and metabolic abnormalities, such as insulin resistance and T2DM [45]. In acute studies, oral administration of high-fat fed rats with *A. squamosa* together with a glucose load improved glucose tolerance with enhanced plasma insulin responses. These results were consistent with the in vitro observations and encouraged the longer- term studies looking at effects of twice daily dosing with *A. squamosa* extract in high-fat fed rats. This revealed significant improvements in non-fasting blood glucose, glucose tolerance, plasma insulin, food, fluid and energy intake.

Several pharmacological approaches have been made to treat T2DM by targeting the incretin system, including development of oral DPPIV inhibitors to prevent GLP-1 degradation [46]. In our studies, incubation with *A. squamosa* extract resulted in significant concentration-dependent inhibition of DPP-IV enzyme activity which, although less potent than sitagliptin, translated to readily demonstrable acute enzyme inhibitory effects in high-fat fed rats. In our longer-term studies, administration of plant extract similarly suppressed plasma DPP-IV enzyme activity suggesting that part of metabolic improvement might involve potentiation of GLP-1 and GIP actions.

More detailed evaluation of these longer-term pancreatic actions of *A. squamosa* revealed reversal of the high-fat fed induced increases in pancreatic insulin content, islet size, beta cell area and alpha cell area in high-fat fed rats. The number of small size islets increased significantly. However, the number of large and medium size islets decreased. There was also a significant reduction in the number of islets per mm^2^. Pancreatic insulin content decreased and so did the area of the islet and beta cells together with a tendency towards decreased numbers of beta cells. The most plausible explanation for these effects of *A. squamosa* is the alleviation of insulin resistance caused by high-fat feeding which increases pancreatic insulin demand. Additionally, the restoration of normal blood glucose control can be envisaged to diminish the direct beta cell effects of *A. squamosa* due to their glucose dependency.

Phytochemical isolation of insulinotropic constituents of *A. squamosa* was performed to structurally identify some of the main compounds responsible for the antidiabetic properties. RP-HPLC fraction isolated as peak-2 revealed a molecular mass of 610 Da which had the same molecular mass and retention time as commercially available rutin. The spectrum obtained of ^1^H NMR displayed characteristic proton signals at 7.57, 6.78, 6.30, 5.11 and 5.01 PPM. In contrast, five aromatic protons were observed in the spectrum of ^1^H NMR; ortho-coupling protons were detected at 7.57 (dd, 1H, *J* = 2.4 Hz, *J* = 8.4 Hz) and 6.78 (d, 1H, *J* = 8.4 Hz) and meta-coupling protons at 6.11 (s, 1H) and 5.01 (s, 1H). The ^1^H NMR spectrum also endorsed glucose and rhamnose moieties at 4.74 ppm with the glucose anomeri proton signal and 4.42 ppm with the rhamnose signal. At 1.18 ppm (d, 3H, *J* = 6.6 Hz), a doublet of rhamnose methyl group was observed at a high field. The remaining protons resonated between 3.37 and 3.21 ppm in the sugar moiety. A doublet of methyl group of rhamnose was also observed at high field at 1.18 (d, 3H, *J* = 6.6 Hz). These features identify the peak as rutin with a chemical structure of C_27_H_30_O_16_. Rutin metabolites, isoquercitrin and quercetin, were also detected in the insulin-releasing peaks confirming earlier studies of flavonoids present in *A. squamosa* [47]. Further studies using rutin isolated from the *A. squamosa* extract, revealed that the compound dose-dependently stimulated insulin release from BRIN-BD11 cells and improved both in vivo glucose tolerance and plasma insulin responses. Other studies have also suggested that both rutin and isoquercitrin, inhibit DPP-IV enzyme activity by directly blocking the DPP-IV active binding site [48,49].

Interestingly, flavonoids such as rutin have been shown previously to exert antihyperglycaemic effects in STZ-induced diabetic rats [50]. This was associated with inhibition of α-glucosidase activity [51] and increased insulin secretion from rat pancreatic islets [52]. Rutin is widely distributed in edible plants [53] can be biotransformed into quercetin and isoquercitrin [54,55] which may have better antidiabetic properties. Thus, quercetin is absorbed in small intestine at a faster rate than rutin [53], yielding higher plasma concentrations and greater bioavailability in man [53]. These observations focus attention on further studies evaluating the therapeutic actions of flavonoids in diabetes.

## 5. Conclusions

In summary, this research has demonstrated that *A. squamosa* plant extract exhibits clear antidiabetic properties mediated by broad spectrum of actions, including enhancing insulin secretion/action, inhibiting starch digestion and protein glycation, delaying glucose absorption and suppressing DPP-IV enzyme activity. Several phytochemicals are likely to contribute to such effects but rutin isolated by bioassay led purification appears to be the major insulinotropic agent. These studies indicate the suitability of *A. squamosa* as a simple multi-purpose dietary adjunct for treatment of T2DM and as a source of antidiabetic agents.

## Abbreviations

ADA	American diabetes association
AUC	Area under the curve
cAMP	Cyclic adenosine monophosphate
CE	Cellulose ester
DAPI	4, 6-diamidino-2-phenylindole
DPP-IV	Dipeptidyl peptidase-IV
EI-MS	Electron ionization mass spectroscopy
GIP	Glucose-dependent insulinotropic polypeptide
GLP-1	Glucagon-like peptide-1
GLUT-4	Glucose transporter type 4
GLY-PRO-AMC	Gly-Pro-7-Amino-4-Methyl-Coumarin
HDL	High-density lipoproteins
HWAS	Hot water extract of Annona squamosa
IBMX	Isobutylmethylxanthine
KATP	ATP-sensitive potassium channel
KCL	Potassium chloride
LC-MS	Liquid chromatography mass spectrometry
LDH	Lactate dehydrogenase
LDL	Low-density lipoprotein
NaBH3CN	Sodium cyanoborohydride
NaCl	Sodium chloride
2-NBDG	2-(N-(7-Nitrobenz-2-oxa-1,3-diazol-4-yl) Amino)-2-Deoxyglucose
NMR	Nuclear Magnetic Resonance
RP-HPLC	Reverse phase high performance liquid chromatography
STZ	Streptozotocin
T2DM	Type 2 diabetes mellitus
TFA	Trifluoroacetic acid
UARC	University Ayurvedic Research Centre
